# Neuroprotective potential of sevoflurane against isoflurane induced cognitive dysfunction in rats via anti-inflammatory and antioxidant effect

**DOI:** 10.1590/acb385523

**Published:** 2023-12-01

**Authors:** Yi Gong, Peipei Kang, Junhui Wang, Yan Chen, Zhongliang Wei

**Affiliations:** 1 Xiamen University – School of Medicin – Department of Anesthesiology – Xiamen ( Fujian), China; 2 Nantong Tumor Hospital – Department of Anesthesiology – Nantong (Jiangsu), China; 3 Taizhou Bo-ai Hosptial – Department of Anesthesiology – Taizhou (Zhangjiang), China.; 4 Xi’an Fourth Hospital – Department of Anesthesiology – Xi’an (Shaanxi), China; 5 Affiliated Hospital of Youjiang Medical University for Nationalities – Department of Anesthesia – Baise (Guangxi), China

**Keywords:** Sevoflurane, Isoflurane, Cognitive Dysfunction, Oxidative Stress, Inflammation

## Abstract

**Purpose:**

Intravenous anesthetics have excellent analgesic activity without inducing the side effect in the respiratory system. The aim and objective of the current experimental study was to access the neuroprotective effect of sevoflurane against isoflurane induced cognitive dysfunction in rats.

**Methods:**

Isoflurane was used for induction the neurodysfunction in the rats, and rats received the oral administration of sevoflurane (2.5, 5 and 10 mg/kg). Morris water test was carried out for the estimation of cognitive function. Neurochemical parameters, antioxidant parameters and pro-inflammatory cytokines were also estimated.

**Results:**

Sevoflurane significantly (P < 0.001) altered the neurochemical parameters such as anti-choline acetyltransferase, acetylcholine esterase, acetylcholine, protein carbonyl, choline brain-derived neurotrophic factor, and amyloid β; antioxidant parameters such as glutathione, superoxide dismutase, and malondialdehyde; pro-inflammatory cytokines include interleukin (IL-2, IL-10, IL-4, IL-6, IL-10, IL-1β), and tumor necrosis factor-α. Sevoflurane significantly reduced the activity of caspase-3.

**Conclusions:**

Sevoflurane exhibited the neuroprotection against the cognitive dysfunction in rats via anti-inflammatory and antioxidant mechanism.

## Introduction

Alzheimer’s disease is the most common form of dementia. As part the reports, approximately 18 million people are infected from the Alzheimer’s disease worldwide, and this figure is almost going to double, approximately 34 million, in 2025^1^^,^ 2. Alzheimer’s disease is considered a neurodegenerative dysfunction categorized via confirmation of amyloid plaques present in the brain in the form of fibrillar proteins^3^. It induces the neurobehavioral abnormalities such as alteration of personality, visual skills, behavioral abnormality, dementia, cognitive dysfunction, and social deterioration^3^. Pathophysiological, neurofibrillary tangles, and senile plaques are situated in the brain compartments (intracellular and extracellular), which induce the atrophy in the synapses and neurons^3^^,^ 4.

During the Alzheimer’s disease, the generation of β-Amyloid (Aβ) in the brain regions (hippocampus and cerebral cortex) starts. Aβ activates the inflammatory process during the Alzheimer’s disease condition, which begins the loss of brain tissue and ultimately induces the neuronal impairment^1^^,^ 2. Inflammation and neurodegeneration are two processes that are thought to be closely linked. Inflammation in the brain is believed to contribute to the degeneration of neurons, which is a key feature of Alzheimer’s disease.

Cytokines such as interleukin (IL)-1β, IL-6 and tumor necrosis factor (TNF)-α are molecules that are produced by immune cells and can contribute to the inflammatory response. Elevated levels of these cytokines have been observed in the brains of individuals with Alzheimer’s disease, and it is thought that they may play a role in the neurodegenerative process. However, the precise mechanisms by which inflammation and neurodegeneration are linked in Alzheimer’s disease are not fully understood, and more research is needed to fully understand the role of these cytokines and other factors in the development and progression of the disease^5^.

Various researchers used isoflurane inducing neurodegeneration model, but this exact model of neurodegeneration mechanism is still unknown. Literature suggests that neuroinflammation plays a critical role in the neurodegeneration of isoflurane induced Alzheimer’s disease model^6^. Neuroinflammation activates the microglial cells and also takes part of astrocytes and neurons. Currently research suggests that the cytokines boost the neuroinflammation and damage of cognitive function^7^.

Cyclooxygenase (COX) plays a significant role in this model^8^. COX related neurodegeneration is crucial for understanding the degeneration mechanism in isoflurane induced Alzheimer’s disease model^9^^,^ 10. It is well established that inflammation plays a role in the development and progression of Alzheimer’s disease, and activated microglia (immune cells in the brain) are thought to contribute to this process through the production of pro-inflammatory cytokines such as IL-1, IL-6, and TNF-α.

While cholinesterase inhibitors, such as donepezil, are commonly used to treat Alzheimer’s disease, they do not address the underlying inflammatory processes that contribute to the disease. On the other hand, non-steroidal anti-inflammatory drugs (NSAIDs) and COX-2 inhibitors, which can reduce inflammation, have shown some promise as a treatment for Alzheimer’s disease, although they can have side effects, including gastrointestinal disturbance and liver and renal toxicity. It seems that our study is attempting to examine the potential of targeting inflammation to treat Alzheimer’s disease in a rodent model^10^^–^ 12. In this study, we tried to scrutinize the neuroprotective effect of sevoflurane against the isoflurane induced neurodegeneration in rats.

## Methods

### Chemical

Sevoflurane (99%) was purchased from the Sigma Aldrich (St. Louis, MO, United States of America). Pro-inflammatory cytokines include TNF-α, IL-2, IL-10, IL-4, IL-6, IL-10, and IL-1β; antioxidant parameters such as glutathione (GSH), malondialdehyde (MDA), and catalase (CAT); and apoptosis marker viz., caspase-3 kit were bought from the Nanjing Jiancheng Bioengineering Institute (Nanjing, China).

### Animal

Sprague-Dawley rats weighed 180–220 g and were 3 months old. Male were obtained from a laboratory animal center and maintained in laboratory conditions with temperature of 22 ± 2°C, 12-hour light-dark cycle, and relative humidity of 60–70%. These animals had unrestricted access to food and water and were treated according to guidelines for the care and use of laboratory animals. The whole study was approved from the Youjiang Medical University (2728392).

*Isoflurane model*

The experimental rats were divided into five groups (six rats in each group), as follow:

- Group A: Normal;
- Group B: Isoflurane control;
- Group C–E: Isoflurane + sevoflurane (2.5, 5 and 10 mg/kg).

In this experiment, rats in groups B–E received infusions of either isoflurane or artificial cerebrospinal fluid into their intracerebroventricular space using a surgical procedure. After inducing anesthesia with isoflurane, the rats were used to assess neurobehavioral and neurochemical parameters. The skulls of the rats were surgically fixed in a stereotaxic device, and a cannula was inserted. To prevent infection, the surgical procedure was performed under sterile conditions. The rats received a special diet, and gentamicin was used to prevent the sepsis. After the surgery, the experimental protocol was initiated^7^.

### Behavioral experimental study

#### Morris water method

The purpose of the Morris water test was to evaluate the learning and memory abilities in rats via using previously published methods with slight modifications^1^^,^ 2.

#### Probe trial

To assess the probe trial, on the final day of the training period, the platform was replaced with a pool, and the experimental rats were allowed to swim freely in the pool for 2 minutes. The time of rats spent in each quadrant was recorded and compared among the groups^7^.

#### Passive avoidance paradigm

The passive avoidance paradigm model was employed to measure the learning and memory abilities of the experimental rats, with slight modifications to previously published methods^1^^,^ 2.

### Neurochemical parameters

Brain-derived neurotrophic factor (BDNF) and amyloid-β peptide were estimated via using the enzyme-linked immunoassay (ELISA) kits, and neurochemical parameters including protein carbonyl, acetylcholinesterase (AchE), acetylcholine (Ach), and acetyltransferase (ChAT) were scrutinized via using the ELISA kits (Nanjing Jiancheng Co., Nanjing, China) according the manufacture’s instruction.

### Antioxidant parameters

GSH, MDA, CAT, and superoxide dismutase used the ELISA kits following the manufacture’s instruction (Nanjing Jiancheng Co., Nanjing, China).

### Cytokines

TNF-α, IL-2, IL-10, IL-4, IL-6, IL-10, and IL-1β (pro-inflammatory cytokines) were measured using commercially available ELISA kits according to the manufacturer’s instructions.

### Caspase-3 activity

Caspase-3 activity in the hippocampus region of the rat brain was determined using commercially available ELISA kits according to the manufacturer’s instructions (Nanjing Jiancheng Co., Nanjing, China).

### Statistical analysis

All the results in the current experimental protocol were showed as mean ± standard error means (SEM) and estimated via one-way analysis of variance (ANOVA) following the Tukey’s test. P < 0.05 was considered as the significant.

## Result

### Morris water test

**Figure 1:**
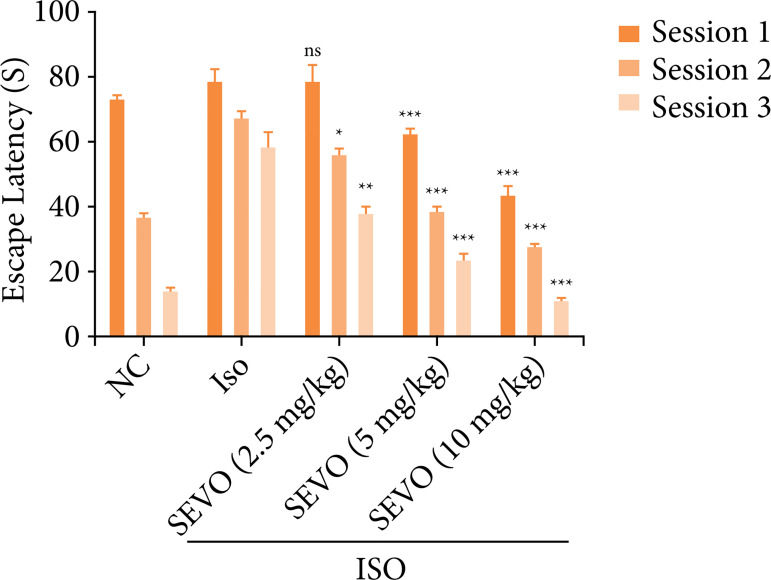
Effect of sevoflurane on isoflurane induced memory and spatial learning in rats determined via using the Morris water maze test. The results are exhibited as mean ± standard error of means. Results obtained are significantly different from the isoflurane-treated group.

Morris water maze test was used to assess the memory and learning abilities of rats. The results showed that the normal rats were able to find the platform more quickly than the rats in the isoflurane group. The isoflurane group had a longer escape latency of 78 seconds compared to the other groups. Among all the experimental groups, the normal rats had the shortest escape latency, followed by the rats treated with sevoflurane (10 mg/kg). These results suggest that sevoflurane may improve memory dysfunction in rats. The sevoflurane-treated rats were able to find the hidden platform in the water pool more quickly and had a shorter escape latency overall (Fig. 1). This suggests that sevoflurane may alter behavioral dysfunction in rats.

*P = 0.05; **P = 0.01; ***P = 0.001; ISO: isoflurane; NC: normal; SEVO: sevoflurane. Source: Elaborated by the authors.

### Passive avoidance paradigm

**Figure 2:**
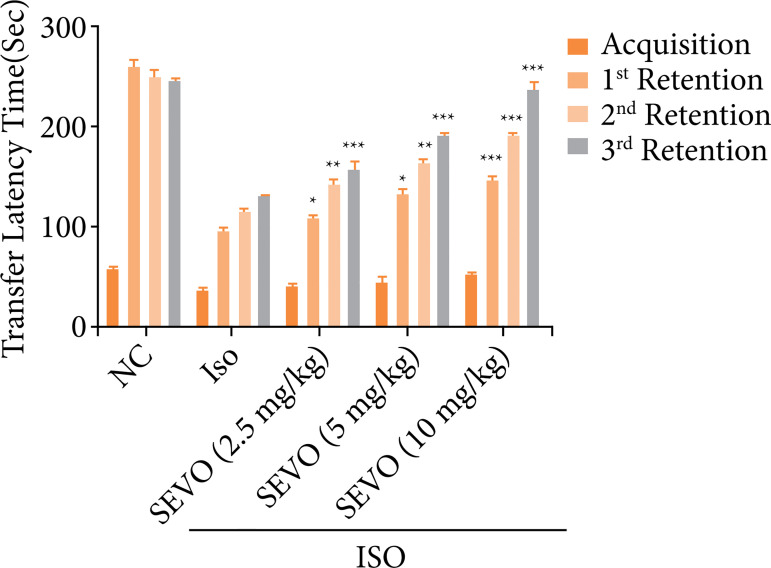
Effect of sevoflurane on isoflurane induced memory and spatial learning in rats determined via using the passive avoidance response. The whole results are exhibited as mean ± standard error of the mean. Results obtained are significantly different from the Iso-treated group.

The passive avoidance paradigm was used to assess the memory and learning abilities of rats. The results showed that the isoflurane group rats had a reduced transfer latency time compared to the normal rats in the acquisition trial. However, in all of the retention trials, both the isoflurane group rats and the sevoflurane-treated group rats had an increased transfer latency time (as shown in Fig. 2). This suggests that both isoflurane and sevoflurane may affect memory and learning in rats.

*P = 0.05; **P = 0.01; ***P = 0.001. Source: Elaborated by the authors.

### Platform quadrant time

**Figure 3:**
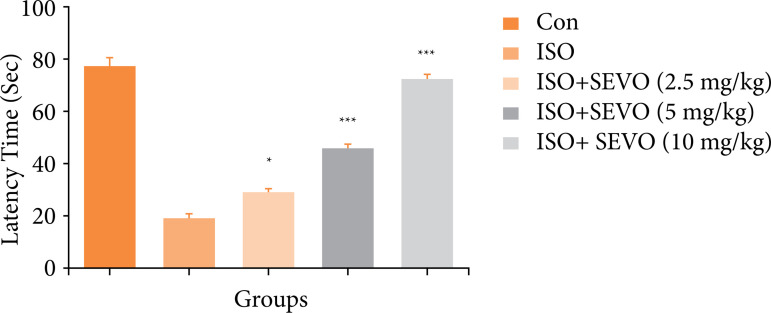
Effect of sevoflurane on isoflurane induced memory and spatial learning in rats determined via using the latency time. The whole results are exhibited as mean ± standard error of the mean. Results obtained are significantly different from the Iso-treated group.

The probe trial was used to measure the time that the rats spent on the platform quadrant. The results showed that the rats in the isoflurane group had a shorter latency time of 19 seconds in the probe trial compared to the other groups. On the other hand, the sevoflurane-treated group rats had an increased latency time in a dose-dependent manner (Fig. 3). This suggests that isoflurane may reduce the time that rats spend on the platform quadrant, while sevoflurane may increase the time that rats spend on the platform quadrant.

*P = 0.05; **P = 0.01; ***P = 0.001. Source: Elaborated by the authors.

### Acetylcholine, acetylcholinesterase, choline acetyltransferase and protein carbonyl

**Figure 4:**
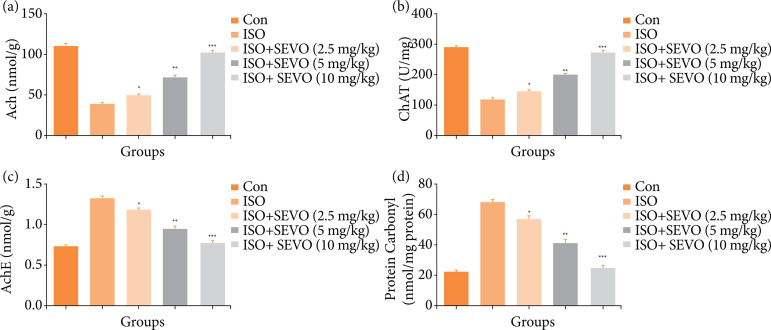
Effect of sevoflurane on isoflurane induced memory and spatial learning in rats determined via estimation of neurochemical parameters. **(a)** Ach, **(b)** ChAT, **(c)** AchE and **(d)** protein carbonyl. The whole results are exhibited as mean ± standard error of the mean. Results obtained are significantly different from the Iso-treated group.

It has been well established that the cholinergic system is important for cognitive function. In cases of cognitive impairment, decreased levels of Ach, AchE and increased levels of protein carbonyl and ChAT have been observed. Similar results were seen in the group of rats treated with isoflurane. In contrast, the group of rats treated with sevoflurane showed increased levels of Ach and AchE and decreased levels of ChAT and protein carbonyl in the hippocampus region of the brain (Fig. 4).

*P = 0.05; **P = 0.01; ***P = 0.001. Source: Elaborated by the authors.

### Amyloid-β peptide and brain-derived neurotrophic factor

**Figure 5:**
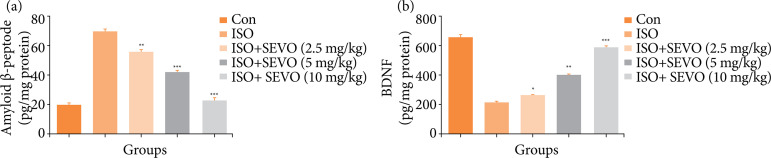
Effect of sevoflurane on isoflurane induced memory and spatial learning in rats determined via estimation of neurochemical parameters. **(a)** Amyloid-β peptide and **(b)** brain-derived neurotrophic factor. The whole results are exhibited as mean ± standard error of the mean. Results obtained are significantly different from the Iso-treated group.

During cognitive impairment, it is common to see a decrease level of amyloid-β peptide and an increase level of BDNF. The isoflurane control group showed the opposite trend, with an increase in the level of amyloid-β peptide and a decrease in the level of BDNF compared to the treated and normal groups. Sevoflurane was found to decrease the level of amyloid-β peptide and increase the level of BDNF in a dose-dependent manner (Fig. 5).

*P = 0.05; **P = 0.01; ***P = 0.001. Source: Elaborated by the authors.

### Antioxidant parameter

**Figure 6:**
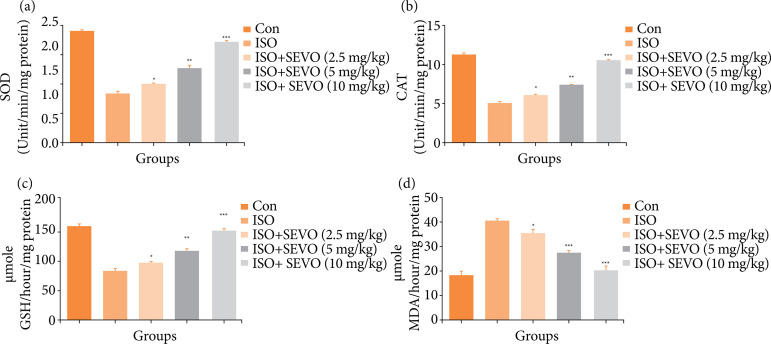
Effect of sevoflurane on isoflurane induced memory and spatial learning in rats determined via estimation of antioxidant parameters. **(a)** SOD, **(b)** CAT, **(c)** GSH, and **(d)** MDA. The results are presented as mean ± standard error of the mean. Statistical analysis indicated that the results for the sevoflurane-treated group were significantly different from those for the isoflurane-treated group, with *P < 0.05, **P < 0.01, and ***P < 0.001 indicating increasing levels of significance.

SOD: superoxide dismutase; CAT: Catalase; GSH: glutathione; MDA: malondialdehyde. Source: Elaborated by the authors.

Oxidative stress is an important factor in the development of cognitive impairment. During cognitive impairment, the balance of endogenous antioxidants is disrupted due to the progression of the disease. The isoflurane group of rats showed a decrease level of CAT, SOD and GSH, and an increase level of MDA. In contrast, rats treated with sevoflurane showed increased levels of CAT, SOD, and GSH and a decrease in the level of MDA (Fig. 6).

### Pro-inflammatory cytokines

**Figure 7:**
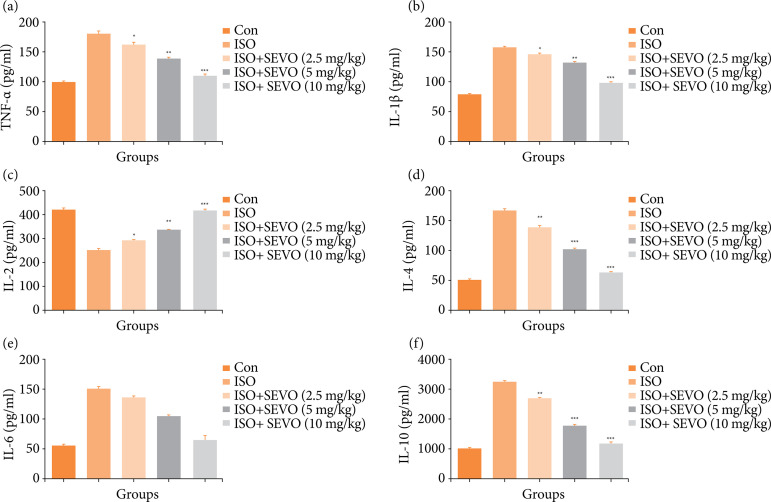
Effect of sevoflurane on isoflurane induced memory and spatial learning in rats determined via estimation of proinflammatory cytokines parameters. **(a)** TNF-α, **(b)** IL-1β, **(c)** IL-2, **(d)** IL-4, **(e)** IL-6 and **(f)** IL-10. The whole results are exhibited as mean ± standard error of the mean. Results obtained are significantly different from the Iso-treated group.

Figure 7 shows the effect of isoflurane and sevoflurane on the levels of pro-inflammatory cytokines. The isoflurane-treated rats showed an increase level of TNF-α, IL-1β, IL-4, IL-6, IL-10 and a decrease in the level of IL-2 compared to the other groups. In contrast, the rats treated with sevoflurane had decreased levels of TNF-α, IL-1β, IL-4, IL-6, IL-10 and increased levels of IL-2 compared to the isoflurane control group rats.

*P = 0.05; **P = 0.01; ***P = 0.001; TNF: tumor necrosis factor; IL: interleukin. Source: Elaborated by the authors.

### Caspase-3 activity

**Figure 8:**
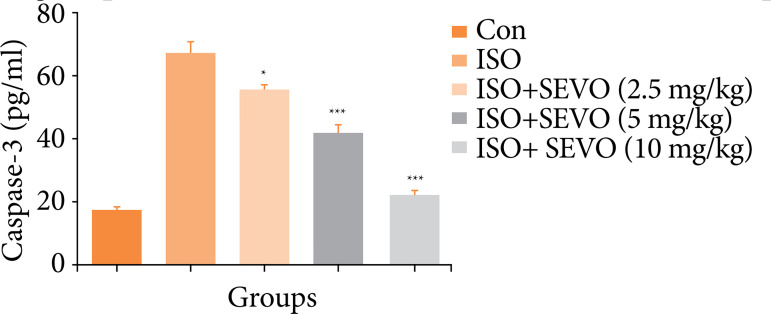
Effect of sevoflurane on isoflurane induced memory and spatial learning in rats determined via estimation of caspase-3 activity. The whole results are exhibited as mean ± standard error of the mean. Results obtained are significantly different from the Iso-treated group.

Figure 8 showed the activity of caspase-3 in different group of rats. Isoflurane control rats exhibited the increased activity of caspase-3, and sevoflurane treated group rats demonstrated the reduced level of caspase-3.

*P = 0.05; **P = 0.01; ***P = 0.001 Source: Elaborated by the authors.

## Discussion

Alzheimer’s disease is the neurodegeneration disease described via deterioration of cortical neurons and hippocampal region in the brain which further starts the injury in memory and induces the cognitive impairment^13^^,^ 14. According to previous research, there are currently an estimated 35.6 million cases of dementia worldwide, and these figures are expected to reach 65.7 million by 2030 and 115.4 million by 2050. Studies also suggest that approximately two-thirds of people living with dementia reside in low- and lower-middle-income countries, as classified by the World Health Organization^7^.

Alzheimer’s disease is a progressive brain disorder that affects memory and cognitive functions. It is the most common cause of dementia in older adults and is characterized by the degeneration of brain cells, leading the reduction in the memory and other cognitive functions. The most common symptoms of Alzheimer’s disease include difficulty with memory, particularly with new information, difficulty with language and communication, difficulty with problem-solving and decision-making, difficulty with spatial orientation, and a decline in overall cognitive function. These symptoms typically worsen over time as the disease progresses^15^^–^ 18.

Isoflurane is commonly used in inhaled anesthetic, and various published reports suggest that the isoflurane induces the detrimental effect especially on the cognitive function in the rats^14^^,^ 19. Neuroinflammation plays an important role for the induction of cognitive impairment after the post-surgery and post-anesthesia^13^. Pathophysiological study suggests that the anesthesia exhibits effect on the neuroinflammation and the researcher targeting the inflammatory pathway to minimize the effect of isoflurane^13^^,^ 20 ^,^ 21. Previous studies showed that the continuous exposure of isoflurane (2%) for 4 hours cause the impairment in the cognitive function^14^. Moreover, in this experimental study, we used the isoflurane model to estimate the neuroprotective effect of sevoflurone.

It is also confirmed that AChE and inflammatory reaction play a significant role in the pathogenesis of Alzheimer’s disease^16^^,^ 17. Both the parameters boosted during the Alzheimer’s disease, due to continuous generation of free radical, and also induced the oxidative stress. Based on previous research, antioxidant and inflammatory therapy are the best approaches to treat the Alzheimer’s disease^14^^,^ 18.

During the Alzheimer’s disease, memory impairment and spatial learning are the common changes in the hippocampus area of brain. Ach is considered as the significant transmitter in brain and contributes to signaling the memory and learning processes^17^. It is well known that AchE is necessary for nervous system to perform the normal function; it quickly finishes the Ach action. ChAT also takes part in the synthesis of Ach, and previous study demonstrated that during the cognitive impairment the ChAT level is reduced and the loss of cholinergic neurons starts^22^. Isoflurane induced rats showed the increased activity of AchE and reduced activity of ChAT and Ach level in the hippocampus region of rat brain, while sevoflurane significantly attenuated these alterations^14^. The result suggests the sevoflurane improved the cognitive impairment after the isoflurane treatment may be involved in the activity of Ach, ChAT and AchE.

According to the previous research, oxidative stress in the brain tissue plays an important role to induce the cognitive impairment^14^^,^ 22. Endogenous antioxidant includes GSH-Px, and SOD protects the tissue against the cellular protection against the oxygen derived free radicals induced injury. First line endogenous antioxidant system SOD converts the superoxide radical into the hydrogen peroxide and after that it is metabolized via GSH-Px, which is considered as the second line of oxidative stress marker^7^. MDA is the indicator of lipid peroxidation during the oxidative stress. In the current experimental study, isoflurane induced group rats showed the increased level of MDA and reduced level of GSH-Px and SOD and sevoflurane significantly (P < 0.001) reduced the MDA level and boosted the level of GSH-Px and SOD^23^. Isoflurane treatment increased the level of MDA suggesting the increased level of lipid peroxidation and induced the localization injury, as well as enhanced the generation of free radicals^23^^,^ 24. Moreover, sevoflurane treatment ameliorates this dysfunction. These results indicate that sevoflurane reduced the isoflurane induced cognitive dysfunction may be related with the suppression of hippocampal oxidative stress.

It is well documented that T lymphocytes play an important role in the immune system^25^. For maintaining the effective body immune system, it is necessary concentration of homeostasis. T lymphocytes such as Th1 and Th2 play a consider role to maintain the balance of immune system. Imbalance occurred due to activation or secretion of inflammatory cells^25^^,^ 26. Few investigations suggest that during the induction of cognitive dysfunction the long-lasting effects to stress response are exhibited. IL-1β secretes during the injury and infection of tissue and suggests the inflammatory states, which proof the different physiological effects^18^^,^ 27.

The results of this study showed that the expression of TNF-α, IL-1β, and IL-6 in the hippocampus of aged rat was elevated following exposure to isoflurane, a finding that aligns with the findings of previous research. Sevoflurane is a general anesthetic that is commonly used during surgery to induce and maintain anesthesia. It is known to have some cognitive side effects, including impaired memory, and learning abilities, particularly in older individuals. These effects are generally temporary and resolve once the individual has recovered from the anesthesia^18^. These findings emphasized that hippocampal inflammatory response might be the mechanism for cognitive impairment after isoflurane exposure. Pro-inflammatory plays a significant role in the expansion of neuroinflammation during the Alzheimer’s disease^18^^,^ 26, and in the current experimental study isoflurane induced rats demonstrated the increase level of cytokines. Pro-inflammatory cytokines like TNF-α boost the cognitive impairment in the brain^28^.

The secretion of TNF-α starts after the exposure of anesthesia and surgery. In addition, TNF-α also increases the level of IL-1β, which increases the cerebral expression of IL-6^20^^,^ 21. The level of IL-1β and IL-6 is increased in the central nervous system. The enhance level of IL-1β in the region of hippocampus exhibits the hindrance of long-term potentiating that causes cognitive dysfunction and finally the impairment of synaptic plasticity^20^^,^ 21. In the current experimental study, isoflurane induced group rats showed the increased level of IL-2, IL-4, IL-10 and reduced level of IL-1β and sevoflurane reversed the cytokines level. Also, sevoflurane significantly reduced the level of cytokines and suggested the anti-inflammatory effect.

## Conclusion

Collectively, we can conclude that sevoflurane had a considerable effect on the improving the isoflurane induced cognitive dysfunction in experimental rats and suggested the neuroprotective effect. The neuroprotective effect can be correlated with sevoflurane excellent capacity to modify the inflammatory mediators, the cholinergic system, and the oxidative system. The result suggests that the sevoflurane might have a beneficial effect to treat the cognitive dysfunction. In future, we will explore the underlying mechanism to estimate the neuroprotective effect.

## Data Availability

The data will be available upon request.
